# Multi-source domain adaptation for EEG emotion recognition based on inter-domain sample hybridization

**DOI:** 10.3389/fnhum.2024.1464431

**Published:** 2024-10-31

**Authors:** Xu Wu, Xiangyu Ju, Sheng Dai, Xinyu Li, Ming Li

**Affiliations:** College of Intelligence Science and Technology, National University of Defense Technology, Changsha, China

**Keywords:** electroencephalogram, emotion recognition, multi-source domain adaptation, sample hybridization, brain computer interaction

## Abstract

**Background:**

Electroencephalogram (EEG) is widely used in emotion recognition due to its precision and reliability. However, the nonstationarity of EEG signals causes significant differences between individuals or sessions, making it challenging to construct a robust model. Recently, domain adaptation (DA) methods have shown excellent results in cross-subject EEG emotion recognition by aligning marginal distributions. Nevertheless, these methods do not consider emotion category labels, which can lead to label confusion during alignment. Our study aims to alleviate this problem by promoting conditional distribution alignment during domain adaptation to improve cross-subject and cross-session emotion recognition performance.

**Method:**

This study introduces a multi-source domain adaptation common-branch network for EEG emotion recognition and proposes a novel sample hybridization method. This method enables the introduction of target domain data information by directionally hybridizing source and target domain samples without increasing the overall sample size, thereby enhancing the effectiveness of conditional distribution alignment in domain adaptation. Cross-subject and cross-session experiments were conducted on two publicly available datasets, SEED and SEED-IV, to validate the proposed model.

**Result:**

In cross-subject emotion recognition, our method achieved an average accuracy of 90.27% on the SEED dataset, with eight out of 15 subjects attaining a recognition accuracy higher than 90%. For the SEED-IV dataset, the recognition accuracy also reached 73.21%. Additionally, in the cross-session experiment, we sequentially used two out of the three session data as source domains and the remaining session as the target domain for emotion recognition. The proposed model yielded average accuracies of 94.16 and 75.05% on the two datasets, respectively.

**Conclusion:**

Our proposed method aims to alleviate the difficulties of emotion recognition from the limited generalization ability of EEG features across subjects and sessions. Though adapting the multi-source domain adaptation and the sample hybridization method, the proposed method can effectively transfer the emotion-related knowledge of known subjects and achieve accurate emotion recognition on unlabeled subjects.

## 1 Introduction

Emotion, as a complex subjective expression of humans, plays a crucial role in daily life, affecting work, learning, memory, and decision-making (Tyng et al., 2017; Alarcao and Fonseca, 2017; Yu et al., 2023). The generation of emotions involves intricate interactions among multiple brain regions, primarily including the prefrontal cortex, temporal lobe, and others, which are essential for the perception, expression, and regulation of emotions (AlShorman et al., 2020). However, emotions can be intentionally or unintentionally suppressed, leading many individuals to struggle with accurately describing their emotional states. This presents significant challenges for analyzing and assessing emotions (Guo et al., 2024).

These challenges highlight the need for accurate and objective emotion recognition, particularly in fields such as human-computer interaction (HCI), healthcare, mental health monitoring, and security. In these domains, utilizing physiological signals for emotion recognition has become an important area of research (Fiorini et al., 2020; Khare et al., 2024). Recent advancements in AI-enabled detection methods have further enhanced the ability to assess emotional states. For instance, AI techniques have been successfully applied to detect anxiety and psychological stress, showcasing their potential to improve emotion recognition performance (Pal et al., 2022; Heyat et al., 2022). Moreover, research has demonstrated a strong correlation between the generation of emotions and the electrical signals produced by cerebral cortex activity, allowing for the distinction of emotional states through signal decoding (Liu et al., 2017; Venkatraman et al., 2017; Malfliet et al., 2017). Electroencephalography (EEG), as a non-invasive physiological signal detection tool, objectively reflects the electrical activity of different brain regions (Parveen et al., 2023). Consequently, numerous studies have employed EEG-based methods for emotion recognition (Ran et al., 2023; Niu et al., 2023).

Nonetheless, the inherent nonstationarity of EEG signals poses significant challenges in EEG-based emotion recognition (Prabowo et al., 2023; Wu et al., 2020). This non-stationarity can cause significant variations in the EEG patterns between different subjects from the same emotional category and even between the same subject at different times, which increases the difficulty of designing effective and robust recognition models. In addition, when traditional machine learning-based methods are used for emotion analysis, the collection and precise annotation of a large amount of EEG data are required. However, limitations of low spatial resolution of the EEG, high noise interference ratio, and long calibration time during data collection make it particularly challenging to train models effectively using large-scale datasets.

Therefore, to alleviate the requirement for large-scale data collection and tedious annotation, an increasing number of studies have leveraged the concept of domain adaptation (DA) to optimize the EEG-based emotion recognition methods (Li W. et al., 2021; Wan et al., 2021). The DA method enables the utilization of labeled data from a source domain to empower predictions in an unlabeled target domain, thereby significantly enhancing learning performance in the target domain. Zheng et al. (2017) have found that there are consistent and stable patterns between different subjects and sessions, which has provided support for the DA implementation into emotion recognition tasks. The application of the DA methods has effectively reduced the need for a large number of labeled samples (Li Y. et al., 2019), pushing the field of EEG emotion recognition toward more efficient and practical directions.

However, in DA-based emotion recognition, the existing methods primarily focus on aligning the marginal distributions of target- and source-domain data, which neglects the risk that the target-domain data of unknown categories might be adapted to incorrect emotional categories, thus preventing effective matching of data with the same emotional category between the source and target domains. Therefore, a more reasonable approach is to reduce the conditional distribution discrepancy between the source domain and the target domain while considering the alignment of the marginal distributions. This will bring the joint distributions of the source and target domains closer together, thus improving the model's decoding performance on target domain data. However, promoting the alignment of conditional distributions between the source and target domains, and achieving effective adaptation for data with the same labels is a challenge.

In order to solve the above problem, guide the target-domain samples to transfer to the correct category, this study constructs the so-called hybrid sample sets and uses it to replace the source domain. The hybrid sample set consists of half the source domain samples and half the hybrid samples, with hybrid samples constructed by linear combination source domain samples with target domain samples that have the highest cosine similarity. These hybrid samples inherit information from both the source and target domain samples and retain the same category labels as the source-domain samples. During the training process, this method allows the model to naturally learn the features of target domain samples. Meanwhile, since the hybrid samples share labels with the source domain samples, they can guide target samples to transfer to their corresponding source-domain samples, which potentially belong to the same category as the target-domain samples. As a result, samples from the same category in both the source and target domains will exhibit similar feature distributions, increasing the probability that the target-domain samples are classified into the correct category, thus effectively achieving conditional distribution alignment between the target and source domains. Additionally, the hybrid sample set retains half of the source domain samples as stable references to help the model maintain a baseline performance.

This study applies this idea to EEG emotion recognition and constructs a sample hybridization-based multi-source DA method, which can achieve excellent performance in different tasks.

The primary contributions of this study can be condensed as follows:

(1) A sample hybridization method is proposed, where each hybrid sample is constructed by hybridizing a sample from the source domain with its most similar sample in the target domain. Hybrid samples incorporate the information from the source and target domain. As training progresses, the model can gradually adapt to the data distribution of the target domain;

(2) A multi-source DA network is designed. The proposed network takes into account the difference in marginal distribution between different domains, and achieves the marginal distribution alignment by using the maximum mean discrepancy (MMD) loss. In addition, a conditional entropy loss is introduced to adapt the feature distribution of the target domain;

(3) The experiments for cross-subject and cross-session emotion recognition are conducted on two publicly available emotion datasets, the SEED and SEED-IV datasets. The experimental results demonstrate the excellent performance of the proposed model.

The subsequent sections of this paper are structured as follows: Section 2 introduces some related works on domain adaptation based emotion recognition. Section 3 describes the details of the materials and methods proposed in this paper. Section 4 presents the results and compares the results with existing methods. Section 5 discusses the proposed method. Finally, Section 6 summarizes this work.

## 2 Related work

In recent years, with the deepening of the analysis and processing of brain electrophysiological signals, the field of affective computing has demonstrated great feasibility, sparking widespread research interest among researchers (Pan et al., 2023). Recent studies aimed to answer the question of the representation of emotions. Currently, there are two widely accepted representation models: the discrete model and the continuous model. Separately, in the discrete model, emotions are categorized into basic emotional states, such as happiness, neutrality, and sadness (Ekman and Friesen, 1971). In the continuous model, emotions are expressed continuously within a three-dimensional space, which is defined by arousal, valence, and dominance (Mehrabian, 1996). In this context, numerous studies have achieved remarkable progress in the field of affective computing using the domain adaptation (DA) method.

For instance, Chai et al. (2016) introduced an innovative subspace-aligned autoencoder (SAAE) that adopts an autoencoder structure capable of performing feature alignment between the source and target domains, enabling the trained classifier to classify emotions in unlabeled data from the target domain effectively. Sun and Saenko (2016) presented an unsupervised DA method to align linear transformations that correspond to the second-order statistics between the target and source distributions, thereby enhancing the generalization capabilities across domains. Wang Y. et al. (2021) designed a prototype-based symmetric positive definite matrix network architecture that can facilitate feature and sample adaptation between distributionally indistinguishable and centroid-aligned subjects. Similarly, Peng et al. (2022) employed the maximum mean discrepancy (MMD) (Borgwardt et al., 2006) method for joint distribution alignment and used graph-based adaptive label propagation for estimating target labels.

With the advancing progress and widespread adoption of deep learning, utilizing deep neural networks for decoding emotion-related EEG signals has emerged as the predominant approach. Li et al. (2018) presented the deep adaptation network (DAN) to address the challenge of generalization in cross-individual emotion recognition. By optimizing the effects of variations across individuals in EEG signals, the DAN can achieve significant performance improvement compared to baseline methods and other DA techniques. Later, Li et al. (2018) proposed a method that integrates adversarial training with associated domain adaptation (ADA) (Li J. et al., 2019) to address domain distribution discrepancy across domains. By enforcing similarity in feature representations between the target and source domains, this approach reduces the impact of domain shift and improves the model's effectiveness when applied to the target domain. Haeusser et al. (2017) introduced a multi-source collaborative adaptation framework, which considers the correlation between domains and features. The authors focused on achieving automatic emotion recognition across topics or datasets using the EEG features. Zhu et al. (2022) incorporated wasserstein adversarial training into the ADA framework within an autoencoder network, aiming to increase the resemblance between marginal and conditional distributions of different domains, ultimately leading to improved performance in domain adaptation tasks. More recently, Wang F. et al. (2021) developed a domain selection method, which could screen the most similar data from the source domain to the new subjects, thereby mitigating the overfitting issue and enhancing the network's generalization capabilities.

Although the above studies have achieved significant results, most of them have primarily concentrated on the overall adaptation between the target domain and multiple source domains while overlooking the potential differences in distributions between various source domains, contributing to the poor generalization ability of the model.

Therefore, many recent studies have attempted to perform differentiated DA between the target domain and multiple source domains, using a common-branch network architecture to achieve one-to-one adaptation between different domains. For instance, Chen et al. (2021) developed a domain adaptation network with a common branch to extract domain-shared, low-level invariant features. In the branch network, they employed the MMD to reduce the differences in marginal distributions between different domains. Similarly, Cao et al. (2022) categorized the EEG data collected from diverse subjects into multiple source domains and incorporated various domain-specific feature extraction modules of differing dimensions within the branch network, thus enabling the extraction of richer and more diverse domain-specific feature representations. She et al. (2023) introduced a joint DA method in the branch network and improved recognition results by calculating domain similarity weights. Although the aforementioned methods are more comprehensive than merging multiple source domains for adaptation, they still lack an efficient method to mitigate the differences in conditional distributions between the target domain and multiple source domains, which can result in poor recognition performance when applied to the target domain.

Aiming to address the mentioned limitation, this paper proposes a sample hybridization-based multi-source DA (SH-MDA) network for EEG emotion recognition. A common-branch network architecture is adopted to achieve one-to-one DA between the target and source domains, and the MMD loss is utilized to measure the marginal distribution differences between different domains. Besides, to reduce the conditional distribution differences between the source and target domains, and considering the significant individual differences between subjects in different source domains, which make it challenging to construct a universal hybrid sample set applicable to all individual's feature distribution, the hybrid sample set is constructed for each source domain and replaces the source domain in model training.

For the construction of hybrid samples within the hybrid sample set, in the field of data augmentation, some data augmentation methods can serve as references. For instance, Zhang et al. (2017) introduced the Mixup method to generate more diverse samples by randomly combining two different samples in each training batch; Zhou et al. (2023) proposed the EEGmixup, which combines EEG data from the same trial to increase the number of samples in both the source and target domains; and, Wang et al. (2024) utilized prior knowledge on the channel distributions and generated new samples by exchanging the left and right hemisphere channels to achieve data augmentation. For simplicity, in our method, we construct hybrid samples by linearly combining the samples with the highest cosine similarity from the source and target domains.

Moreover, to improve the recognition performance of the model on the unlabeled samples of the target domain, this study introduces conditional entropy to make the model's decision boundary more adaptive to the target-domain feature distribution after model processing.

## 3 Materials and methods

**Figure 1:**
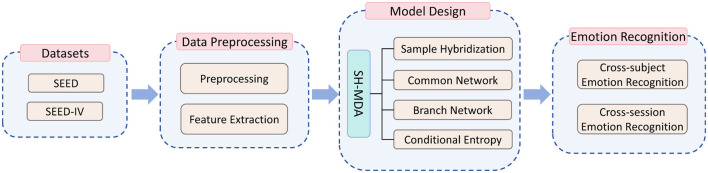
The flowchart of this study.

This study was completed in several steps, as illustrated in Figure 1. These steps include the EEG datasets (utilizing publicly available EEG emotional datasets), data preprocessing, and model design, which details the proposed SH-MDA model. Finally, the proposed model is applied for emotion recognition in cross-subject and cross-session tasks.

### 3.1 Datasets

In this study, the the Shanghai Jiao Tong University (SJTU) emotional EEG dataset (SEED) and its extended version SEED-IV are selected to validate the effectiveness of our proposed method, which are widely used in the field of EEG emotion recognition. The data acquisition and ethical considerations can be seen in Zheng and Lu (2015) and Zheng et al. (2018), all EEG data used in this paper are licensed, and for privacy protection, only numbers are used to represent different subjects. The brief description of the datasets are shown in Table 1.

**Table 1:** The summary of the SEED and SEED-IV datasets.

	**SEED**	**SEED-IV**
Number of subjects	15	15
Number of experiment trials	15	24
The length of EEG data in a trial	4 min	2 min
Emotion category	Positive, neural, negative	Happy, neural, fear, sad
Number of channels	62	62

#### 3.1.1 SEED dataset

The SEED dataset comprises EEG recordings from 15 individuals, consisting of seven males and eight females. It employs video stimuli to evoke corresponding emotions. The video materials were derived from 15 emotional clips selected from movies, each ~4 min in length, with five clips representing each emotion. All subjects participated in three separate experiments conducted at one-week intervals. During each experiment, participants viewed the 15 emotional clips while their brain activity was recorded via EEG. After each video segment, participants had 45 s for self-assessment to ensure the effectiveness of the emotional induction.

#### 3.1.2 SEED-IV dataset

The SEED-IV dataset contains the EEG data of four emotion categories: neutral, sad, fear, and happy. Its structure is similar to that of the SEED dataset; it also includes data from 15 subjects who completed three trials, each comprising 24 experimental trials. In each trial, participants were first presented with a 5-s cue intended to prime their emotional state. Following this cue, they watched a 2-min film segment specifically chosen to evoke the target emotion. After the film, a 45-s self-assessment period was included during which participants rated their emotional experiences.

### 3.2 Data preprocessing

#### 3.2.1 Preprocessing

For the SEED dataset, the downsampling rate for raw EEG signals was set to 200 Hz, and the signals underwent preprocessing using band-pass filtering from 0 to 70 Hz. Subsequently, the EEG data was segmented using non-overlapping time windows, each 1 s in length. Finally, the number of samples per subject per experiment was 3,394.

The SEED-IV dataset utilizes a preprocessing approach similar to that of the SEED dataset, which initially employs a band-pass filtering step to isolate frequencies ranging from 1 to 75 Hz. Subsequently, the EEG data is segmented using a non-overlapping time window of 4-s length. Finally, the sample size for each subject across the three sessions is 851, 832, and 822, respectively.

#### 3.2.2 Feature extraction

In the selection of input features for the model, recent studies have revealed that differential entropy (DE) features can effectively extract emotional information from EEG signals, thereby enhancing the classification performance of a model (Ju et al., 2024; Lu et al., 2023; Li et al., 2023; Liang et al., 2021). For a segment pre-processed time series EEG data *X*, which approximates a Gaussian distribution *N*(μ, σ^2^), DE features can be calculated as follows


(1) $$\begin{array}{l} f\left(X\right)=\int_{-\infty }^{+\infty }\frac{1}{\sqrt{2\pi }\sigma^{2}}e^{-\frac{{\left(x-\mu \right)}^{2}}{2\sigma^{2}}}\log\left(\frac{1}{\sqrt{2\pi \sigma^{2}}}e^{-\frac{{\left(x-\mu \right)}^{2}}{2\sigma^{2}}}\right)dx\\ \;=\frac{1}{2}\log\left(2\pi e\sigma^{2}\right). \end{array}$$

The differential entropy is a simple measure of the time series complexity in a specific frequency band. In this study, for both the SEED and SEED-IV datasets, after dividing samples using different time windows, we extracted DE features for each sample across the δ (1–3 Hz), θ (4–7 Hz), α (8–13 Hz), β (14–30 Hz), and γ (31–50 Hz) frequency bands and normalize them by different channels. Finally, the feature dimension is 62 × 5 (channels × frequency bands).

### 3.3 Model design

#### 3.3.1 The framework of SH-MDA

**Figure 2:**
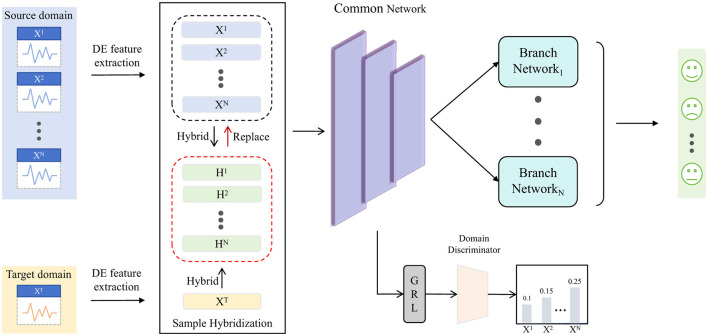
The training process of the proposed framework of SH-MDA. *N* hybrid sample sets are constructed and replace the corresponding source domain. The hybrid sample set consists of half of the source domain samples and half of the hybrid samples. The common network is utilized to extract domain-invariant features, and the domain discriminator is used to strengthen the extraction through adversarial training. The multiple branch networks are employed to extract domain-specific features and achieve one-to-one adaptation.

This study aims to construct an emotion recognition model using the DA method. The training procedure of the proposed SH-MDA model is shown in Figure 2. Suppose there are multiple labeled EEG data from source subjects (domains) and unlabeled EEG data from a target subject (domain), defined as $X^{S}={\left\{X^{i}\right\}}_{i=1}^{N}={\left\{{\left\{x_{j}^{i},y_{j}^{i}\right\}}_{j=1}^{M_{i}}\right\}}_{i=1}^{N}$ and $X^{T}={\left\{x_{j}^{t}\right\}}_{j=1}^{M_{t}}$, where the *N* denotes the number of domains, *M*_*i*_ represents the number of samples in the *i*-th domain. Firstly, after preprocessing, the hybrid sample sets ${\left\{H^{i}\right\}}_{i=1}^{N}$ are constructed and replace the *N* source domains. Each hybrid sample set consist of half source domain samples and half hybrid samples. Then, the common domain-invariant features of the samples are extracted using the common feature extractor in the common network and reinforced through adversarial training with a domain discriminator. Subsequently, these extracted features are passed to different branch networks, which comprise branch feature extractors (BFEs) and branch task classifiers (BTCs). The BFEs extract domain-specific features for each domain and forward them to the BTCs that compute their respective prediction results. Finally, the loss functions are calculated to update the model.

In addition, in the stage of model prediction, the domain-invariant features of target domain samples extracted by common feature extractor are sent to *N* branch networks to obtain *N* probability distributions of emotion categories, and the final emotion recognition result is output by averaging these probability distributions.

#### 3.3.2 Sample hybridization

**Figure 3:**
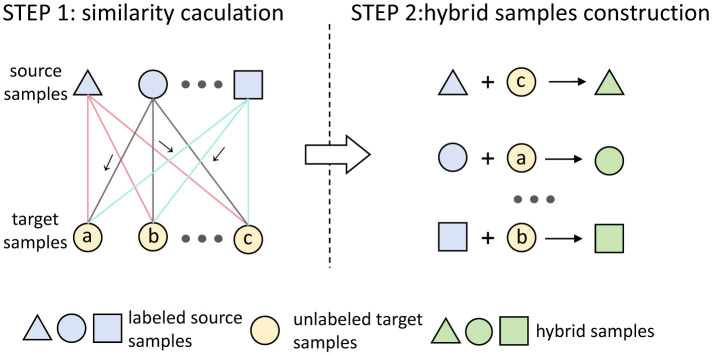
Illustration of sample hybridization, different graphs are used to represent different labels. The symbol “ → ” represent the highest similarity among the source sample and the target sample. The sample hybridization can be divided into two steps. STEP 1: Compute the cosine similarity between the source domain samples and the target domain samples. STEP 2: Linearly combine the source domain samples with their most similar samples in the target domain to construct hybrid samples, which share the same labels as the source domain samples.

Aiming to minimize the conditional distribution difference between source domains and the target domain, this study introduces a conditional alignment method that utilizes sample hybridization, as briefly illustrated in Figure 3.

Specifically, the sample hybridization can be divided into two steps. In the first step, the cosine similarity is used as a measurement to compute the similarity between samples in a given source domain and samples in the target domain. In a training batch, for the sample $x_{j}^{i}\in {\mathbb{R}}^{m\times d}$ from the *i*-th source domain $X^{i,b}={\left\{x_{j}^{i}\right\}}_{j=1}^{M_{b}}$, where *M*_*b*_ denotes the batch size, *m* and *d* represent the number of channels and feature dimension per channel, the *s*_*j, k*_ is defined as the cosine similarity between $x_{j}^{i}$ and the sample $x_{k}^{t}\in {\mathbb{R}}^{m\times d}$ in the target domain $X^{T,b}={\left\{x_{k}^{t}\right\}}_{k=1}^{M_{b}}$, the *s*_*j, k*_ can be calculated as


(2) $$\begin{array}{l} s_{j,k}=\frac{x_{j}^{i}\cdot x_{k}^{t}}{∥x_{j}^{i}∥∥x_{k}^{t}∥}, \end{array}$$

where ∥·∥ denotes the Euclidean norm. Next, the normalized cosine similarity between $x_{j}^{i}$ and *M*_*b*_ samples of the target domain in the training batch can be represented by a vector $D_{j}={\left({\tilde{s}}_{j,k}\right)}_{k=1}^{M_{b}}\in {\mathbb{R}}^{M_{b}}$, and ${\tilde{s}}_{j,k}$ is calculated by


(3) $$\begin{array}{l} {\tilde{s}}_{j,k}=\frac{e^{s_{j,k}}}{\sum_{k^{\prime }=1}^{M_{b}}e^{s_{j,k^{\prime }}}}. \end{array}$$

Furthermore, for the sample $x_{j}^{i}$ in *X*^*i, b*^, by calculating the normalized similarity *D*_*j*_, there is a target domain sample ${\tilde{x}}_{j}^{t}$ with the highest similarity. Theoretically, compared with the other samples in the target domain, sample ${\tilde{x}}_{j}^{t}$ is most likely to belong to the same emotional category as sample $x_{j}^{i}$. However, due to the difference between the target and source domains (subject), there exists a significant risk that if the label of the source domain sample is directly utilized as the pseudo label for its most similar target domain sample to conduct domain adaptation, which might cause irreversible negative transfer and subsequently affect the effectiveness of domain adaptation.

Therefore, in the second step, we construct the hybrid samples through a linear combination of the source-domain samples and their most similar samples in the target domain, while sharing labels with the source-domain samples. This way, during training, these hybrid samples can provide feature information of the target domain samples and be classified into corresponding emotion categories based on the shared labels, enabling the model to gradually adapt to the data distribution of the target domain.

In the above, we introduced the construction process of hybrid samples. Finally, to ensure that the target domain data in the hybrid samples does not overly affect the source domain data and to maintain the dominance of the source domain data, we retain half of the source domain samples when constructing the hybrid sample set. These source domain samples will serve as a stable reference to help the model maintain baseline performance. For the *i*-th source domain, the hybrid sample set $H^{i,b}={\left\{h_{j}^{i}\right\}}_{j=1}^{M_{b}}$ in a training batch can be defined as


(4) $$\begin{array}{l} \;H^{i,b}=\left\{h_{j}^{i},\;j=1,2,\ldots ,M_{b}\right\}\\ h_{j}^{i}=\{\begin{array}{ll} x_{j}^{i} & \text{if }j\leq M_{b}/2,\\ \lambda x_{j}^{i}+(1-\lambda ){\tilde{x}}_{j}^{t} & \text{if }j>M_{b}/2. \end{array} \end{array}$$

where λ is the hybrid parameter, ${\tilde{x}}_{j}^{t}$ represents the sample with the highest cosine similarity to source domain sample $x_{j}^{i}$ in the target domain within the current training batch. Since a hybrid sample set *H*^*i, b*^ is composed of samples from the source and target domains with the highest similarity, which can be considered that samples from the source domain and samples obtained after hybridizing the source-domain samples have a high probability of belonging to the same emotional category. Therefore, the source-domain labels can as the pseudo labels of the hybrid sample set and the hybrid sample sets are defined as $H={\left\{H^{i}\right\}}_{i=1}^{N}={\left\{{\left\{h_{j}^{i},y_{j}^{i}\right\}}_{j=1}^{M_{i}}\right\}}_{i=1}^{N}$.

#### 3.3.3 Common network

In the common network, this study designs a simple three-layer multilayer perceptron (MLP) common feature extractor *E*_*I*_, which aims to extract the domain-invariant features by mapping the input features from the original space to the common feature space, for the samples *h*^*i*^∈ℝ^*m*×*d*^ from *i*-th hybrid sample set *H*^*i*^ and the samples *x*^*t*^∈ℝ^*m*×*d*^ from target domain *X*^*T*^, the domain-invariant features $f_{I}^{i}$ and $f_{I}^{t}$ can be formulated as follows


(5) $$\begin{array}{l} \begin{array}{c} f_{I}^{i}=E_{I}\left(h^{i};\theta_{I}\right)\\ f_{I}^{t}=E_{I}\left(x^{t};\theta_{I}\right) \end{array}, \end{array}$$

where *E*_*I*_(·) represents the common feature extractor in the common network, and θ_*I*_ denotes its parameters. To enhance the capability of the model to extract domain-invariant features, the domain discriminator *D*_*dis*_ is introduced. *D*_*dis*_ consists of a fully connected layer, a softmax layer, and a gradient reversal layer (GRL), and the domain discrimination loss *L*_*dis*_ can be expressed as


(6) $$\begin{array}{l} L_{dis}=\overset{N}{\underset{i=1}{\sum }}E_{h^{i}\sim H^{i}}\left[D^{i}\log\left(D_{dis}\left(f_{I}^{i},\theta_{D}\right)\right)\right], \end{array}$$

where θ_*D*_ represents the parameter of *D*_*dis*_, $f_{I}^{i}$ and *D*^*i*^ denote the domain-invariant features and the domain label of *i*-th hybrid sample set. The optimization objective of the domain discriminator is to induce the common feature extractor to more effectively extract domain-invariant features through adversarial training, by minimizing the value of *L*_*dis*_, the parameters of the common feature extractor are updated by the gradient reversal layer in the direction of the opposite gradient. This makes the extracted features more general.

#### 3.3.4 Branch networks

**Figure 4:**
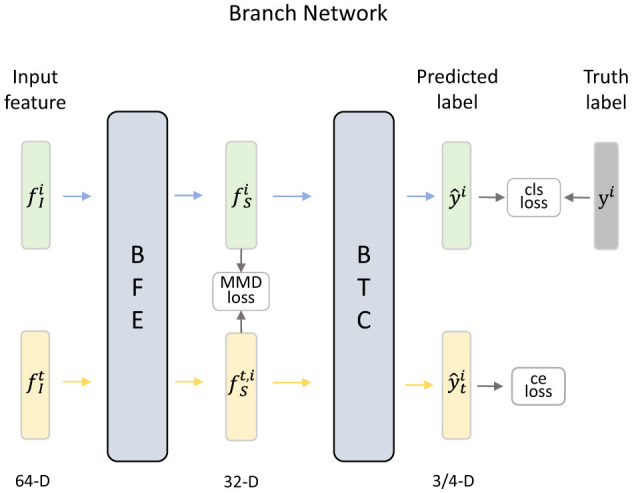
Details of the *i*-th branch network, which can achieve an effective alignment of the distribution between a specific hybrid sample set and the target domain by minimizing the loss functions.

In this paper, we design different branch networks to achieve one-to-one distribution alignment by mapping the features of each source domain and target domain into specific feature spaces. As shown in Figure 4, each branch network consists of a branch feature extractor (BFE) and a branch task classifier (BTC).

Specifically, after extracting domain-invariant features ${\left\{f_{I}^{i}\right\}}_{i=1}^{N}$ and $f_{I}^{t}$, the features of different hybrid sample set and target domain are fed into the corresponding branch network. In the branch network, the BFE employs a fully connected layer to map data into an individual latent space and obtains domain-specific features. In *i*-th BFE $E_{S}^{i}$, the domain-specific features $f_{S}^{i}$ and $f_{S}^{t,i}$ are extracted by


(7) $$\begin{array}{l} \begin{array}{c} f_{S}^{i}=E_{S}^{i}\left(f_{I}^{i};\theta_{S}^{i}\right)\\ f_{S}^{t,i}=E_{S}^{i}\left(f_{I}^{t};\theta_{S}^{i}\right) \end{array}, \end{array}$$

in which $\theta_{S}^{i}$ denotes the parameters of $E_{S}^{i}$. In addition, the Maximum Mean Discrepancy (MMD) loss is calculated to measure the differences in marginal distributions across domains. The formula of *L*_*MMD*_ can be expressed as


(8) $$L_{MMD}=\sum_{i=1}^{N}E_{h^{i},x^{t}\sim H^{i},X^{T}}\left[||\frac{1}{M_{i}}\sum_{j=1}^{M_{i}}f_{S,j}^{i}-\frac{1}{M_{t}}\sum_{k=1}^{M_{t}}f_{S,k}^{t,i}|{|}_{H}^{2}\right],$$

where $f_{S,j}^{i}$ represents the domain-specific feature of the sample in the *i*-th hybrid sample set, $f_{S,k}^{t,i}$ denotes the domain-specific feature of the sample in the *i*-th branch network of the target domain, and *M*_*i*_ and *M*_*t*_ are the numbers of samples in the source domain and target domain, respectively. Then, the extracted domain-specific features of hybrid sample sets ${\left\{f_{S}^{i}\right\}}_{i=1}^{N}$ and target domain ${\left\{f_{S}^{t,i}\right\}}_{i=1}^{N}$ are sent to corresponding BTC to reduce feature dimensions to the number of emotion categories, and the softmax layer translates the output as probability distribution. The prediction of *i*-th BTC can be expressed as


(9) $$\begin{array}{l} \;{\hat{y}}^{i}=E_{C}^{i}(f_{S}^{i};\theta_{C}^{i})\\ {\hat{y}}_{t}^{i}=E_{C}^{i}(f_{S}^{t,i};\theta_{C}^{i}) \end{array}$$

Where $E_{C}^{i}$ represents the *i*-th branch task classifier, ŷ^*i*^ and ${ŷ}_{t}^{i}$ denote the prediction results in *i*-th BTC. Furthermore, the classification loss is used to measure the difference between the predictions and the truth labels. During the training process, the calculation of classification loss *L*_*cls*_ can be expressed by cross-entropy as follows:


(10) $$\begin{array}{l} L_{cls}=\overset{N}{\underset{i=1}{\sum }}E_{h^{i}\sim H^{i}}\left[-\overset{C}{\underset{c=1}{\sum }}p\left(c=y^{i}\right)\log\left({\hat{y}}^{i,c}\right)\right], \end{array}$$

where *p*(·) is the indicator function, ${\hat{y}}^{i,c}$ is the prediction of *i*-th hybrid sample set in *c*-th emotion category, *y*^*i*^ represents the truth label of *i*-th source domain, *C* denotes the number of emotion categories. By minimizing the *L*_*cls*_, the hybrid samples will exhibit a similar conditional distribution to that of the source domain samples. Since the hybrid samples incorporate information from the target domain samples, they can serve as a guide to align these target samples with the source domain samples, enhancing the model's adaptation effect, and improving the classification accuracy on the target domain.

#### 3.3.5 Conditional entropy

In this study, the target domain data are unlabeled. Therefore, after training the classifiers using the data from source domain, the classifier might align more closely with the data distribution of the source domain while paying less attention to that of the target domain. Consequently, the decision boundary of classifier in the target domain could be inaccurate. To address this issue, this study introduces the conditional entropy loss *L*_*ce*_, which is defined by Shu et al. (2018):


(11) $$\begin{array}{l} L_{ce}=-\overset{N}{\underset{i=1}{\sum }}E_{x^{t}\sim X^{T}}\left[\left({\hat{y}}_{t}^{i}\right)\ln\;{\hat{y}}_{t}^{i}\right], \end{array}$$

where ${\hat{y}}_{t}^{i}={\left\{{\hat{y}}_{t,j}^{i}\right\}}_{j=1}^{M_{t}}$ denotes the prediction result of the target-domain data output from *i*-th branch network. The conditional entropy loss measures the classification uncertainty of classifier. A high value of conditional entropy loss indicates that the classifier has a large uncertainty about the category belonging to input data. Therefore, by minimizing the conditional entropy loss, the classifier can be forced to make more certain predictions in the target domain, even though those predictions might not be completely accurate. In this way, the classifier's decision boundary will be pushed away from the high-density regions of the target domain data, reducing the probability of making uncertain predictions in these regions.

In summary, in the training stage, the proposed SH-MDA receives samples from source and target domains, and achieves distribution alignment between the source and target domains. The parameters of SH-MDA can be updated by minimizing the *L*_*total*_ :


(12) $$\begin{array}{l} L_{total}=L_{cls}+\lambda_{1}L_{dis}+\lambda_{2}L_{mmd}+\lambda_{3}L_{ce}, \end{array}$$

where the λ_1_~λ_3_ is the balance parameters. The training procedure of SH-MDA model is presented in Algorithm 1.

**Algorithm 1:**
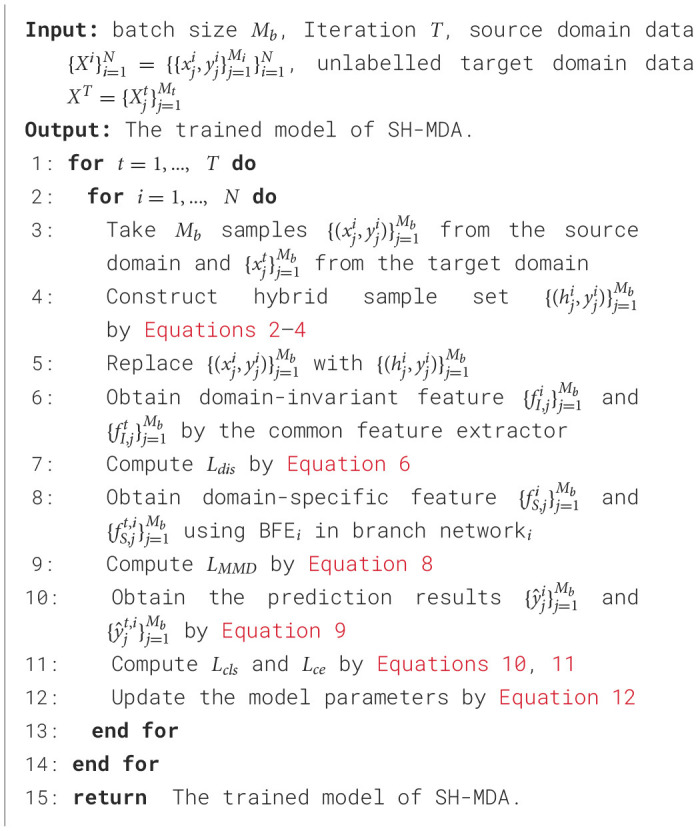
The training procedure of SH-MDA.

### 3.4 Experiments

The proposed SH-MDA is implemented on PyTorch framework version 1.8.1 with the CUDA toolkit version 10.1, conducted cross-subject and cross-session experiments and compared with several state-of-the-art methods on two public emotion datasets. All the experiments were performed on a PC with an Intel (R) Core (TM) i9-10900X CPU, an NVIDIA GTX 2080Ti GPU, running the Windows 10 operating system. The codes of SH-MDA are available at https://github.com/Xitsuka/SH-MDA.

In the experiment, two validation paradigms were adopted: cross-subject and cross-session. In the common feature extractor, the extracted DE features were fed as the model input, and the feature dimension was reduced from 310 (62 × 5) to 64 after three layers of the MLP. In the three-layer MLP, a LeakyRelu (Xu et al., 2015) layer was used after each linear layer. In the domain discriminator, domain-invariant features pass through two fully connected layers, with the dimensionality reduced from 64 to the number of source domains. Next, in the BFE and BTC, only one fully connected layer was used; the BFE reduced the features from 64-D to 32-D, and then the BTC reduced the 32-D features to the number of categories. The Table 2 describes the network hyperparameters of SH-MDA.

**Table 2:** Hyperparameters of proposed SH-MDA.

	**Layer**	**Hyperparameters**
Common feature extractor	Flatten	–
	Fully connected	25,612,864
	LeakyRelu	Negative slope = 0.01
Domain discriminator	Fully connected	32, number of source domain
	LeakyRelu	Negative slope = 0.01
	Gradient reversal layer	λ = 0.8
	Softmax	–
Branch feature extractor	Fully connected	32
	Batch normalization	32, eps = 1e-05
	LeakyRelu	Negative slope = 0.01
Branch task classifier	Fully connected	Number of category
	Softmax	-

In the training process of SH-MDA, the Adam optimizer (Kingma and Ba, 2014) was used to update the network parameters. The balance parameter λ_1_ for *L*_*dis*_ was set to 0.1, selected from {0.01, 0.1, 0.5, 1.0} based on optimal performance. The *L*_*MMD*_ used a dynamic parameter $\lambda_{2}=\frac{2}{1+e^{-10*i/\text{epoch}}}-1$, as utilized in many studies (Chen et al., 2021; She et al., 2023), with its value increasing with the number of iterations. As the number of iterations progressed, the marginal distribution alignment between the source and target domains was gradually achieved. For λ_3_, we followed the setting from Shu et al. (2018); Jiménez-Guarneros and Fuentes-Pineda (2023), using a balance parameter of 0.1. The hybrid parameter λ was searched within the range of {0.4, 0.5, ..., 1.0}, and the optimal value is 0.8 in the cross-subject experiments and 0.6 in the cross-session experiments.

In addition, since the common feature extractor was updated by different domains, and the BFE and BTC received data only from the specific domains, the learning rate of the common feature extractor was set to a small value to ensure model stability. Particularly, the learning rate was set to 5e-4 for the common feature extractor, and 5e-3 for the domain discriminator and branch networks. The hyperparameters setting of experiments is described in Table 3 .

**Table 3:** The parameters setting for the experiments on SEED and SEED-IV datasets.

	**SEED**	**SEED-IV**
Batch size	64	64
Number of categories	3	4
**Learning rate**		
-Common network	5e-4	5e-4
-Else	5e-3	5e-3
Optimizer	Adam	Adam
Number of epoch	50	200

## 4 Results

### 4.1 Results of cross-subject experiments

In the cross-subject experiment, the leave-one-subject-out (LOSO) cross-validation strategy was used to evaluate the effectiveness of the model. In one session, a specific subject served as the target-domain data, while the data from that same session but originating from the other subjects was utilized as the source domain. The process of training and validation was repeated until each subject's sessions had been designated as the target once. For instance, in the SEED dataset, which contains data from 15 subjects, the data from one subject was designated as target-domain data, while the data from the other 14 subjects served as source-domain data, thus, there were 15 tasks in a single session. In this study, the average recognition accuracy of three sessions for each subject was used as the recognition accuracy of that subject, and the average accuracy of all the subjects was used as the final result of the cross-subject experiment of the model. The experiments on the SEED-IV dataset were conducted in the same way.

**Figure 5:**
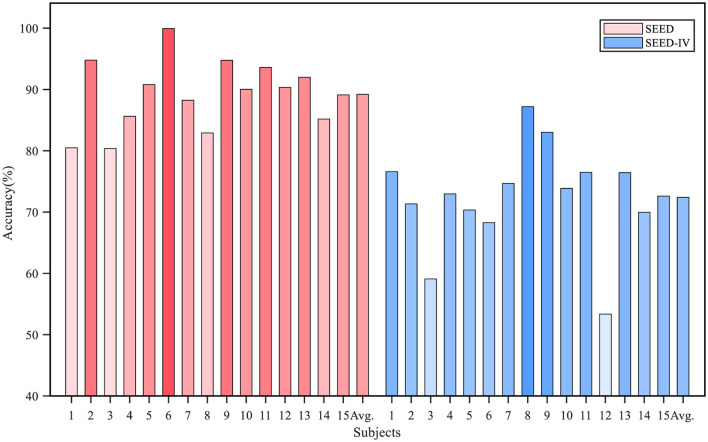
The recognition accuracy in the cross-subject experiment on the SEED and SEED-IV datasets. the *i*-th bar represents the average accuracy when *i*-th subject is selected as target domain.

The accuracy for each subject is shown in Figure 5. The average recognition accuracy of the proposed model for each subject in the SEED dataset exceeded 80%, with a minimum of 80.37% and a maximum of 99.94%. Additionally, the recognition accuracy for eight out of the 15 subjects was higher than 90%. On the SEED-IV dataset, the proposed algorithm achieved a lowest average accuracy of 53.36%, a highest accuracy of 87.19%, and exceeded 70% accuracy for 11 subjects. In addition to accuracy, we analyzed the sensitivity, specificity, and F1 score of the model in cross-subject experiments. The results are presented in Table 4. On the SEED dataset, the sensitivity, specificity, and F1 score were 90.49, 95.71, and 90.24%, respectively; on the SEED-IV dataset, they were 74.29, 91.74 and 73.32%.

**Table 4:** Performance of the propose method in cross-subject experiments.

	**Performance measure**			
**Dataset**	**ACC** ±**STD (%)**	**Sensitivity (%)**	**Specificity (%)**	**F1 score (%)**
SEED	90.27 ± 05.56	90.49	95.71	90.24
SEED-IV	73.41 ± 08.27	74.29	91.74	73.32

### 4.2 Results of cross-session experiments

In the SEED and SEED-IV datasets, each subject conducted the experiment with three sessions. For each subject, in the cross-session experiments, the three sessions were used as the target domain in sequence, with each session serving as the target domain once while the other two served as the source domain. Finally, the average classification accuracy of the three sessions of all subjects was denoted as cross-session experimental result of the model.

**Figure 6:**
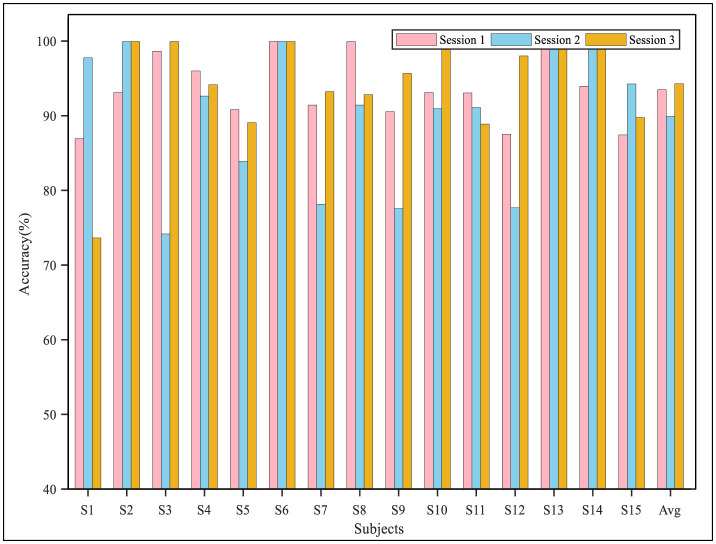
The classification accuracy in the cross-session experiment on the SEED dataset.

**Figure 7:**
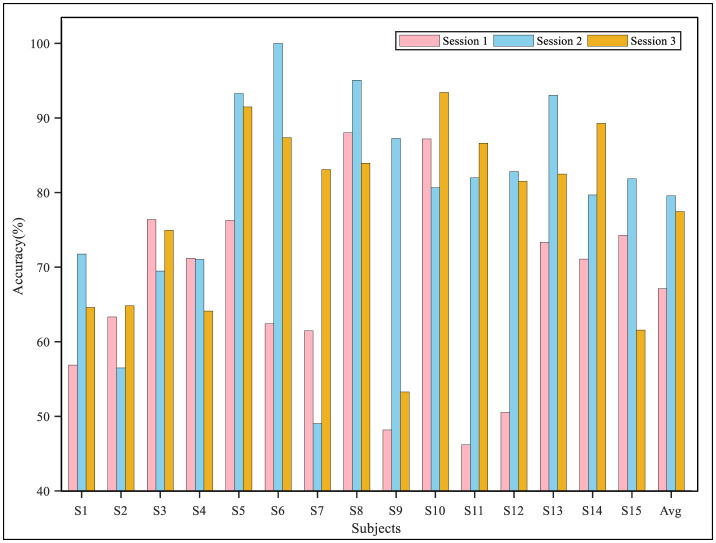
The classification accuracy in the cross-session experiment on the SEED-IV dataset.

The performance of the proposed model in the three sessions for each subject in the two datasets is shown in Figures 6, 7. The results demonstrated that in the cross-session experiment, where both the source and target domain were from the same subject's data, the difficulty of DA was reduced, and thus, the performance was better than that of the cross-subject experiment. On the SEED data set, the average accuracy of the three sessions was higher than 90%, and the accuracy of most sessions was higher than 80%. Meanwhile, the experiment results on the SEED-IV dataset also indicated good performance of the proposed model. The accuracy of the algorithm when the second and third sessions were used as a target domain was significantly higher than that when the first session was used, reaching 80.78 and 76.18%, respectively. Furthermore, as shown in Table 5, we also calculated the performance metrics of the model in cross-session experiments, including sensitivity, specificity, and F1 score. On the SEED dataset, these metrics were 94.18, 96.98, and 94.15%, while on the SEED-IV dataset, they were 75.69, 92.59, and 75.21%.

**Table 5:** Performance of the propose method in cross-session experiments.

	**Performance measure**			
**Dataset**	**ACC** ±**STD (%)**	**Sensitivity (%)**	**Specificity (%)**	**F1 score (%)**
SEED	94.16 ± 06.92	94.18	96.98	94.15
SEED-IV	75.05 ± 08.19	75.69	92.59	75.21

### 4.3 Comparison with the existing methods

To better demonstrate the performance of the model, the proposed method was compared with other optimal methods reported in existing studies. Table 6 shows the recognition accuracy and standard deviation of these methods across subjects in the SEED and SEED-IV datasets. Since the SEED dataset contained three emotion categories and the SEED-IV dataset contained four emotion categories, the classification accuracy of the model on the SEED-IV dataset was lower than that on the SEED dataset. For the SEED dataset, the proposed algorithm was significantly superior to the other algorithms, with an average accuracy of 90.27% in the three sessions, which was about 2% higher than that of the currently optimal algorithm. Meanwhile, the standard deviation of the proposed method was 5.56, which was also at the level of the existing best-performing method, demonstrating the stability of the proposed method. On the SEED-IV dataset, the proposed SH-MDA also showed better performance than the other methods, with an average accuracy of 73.41%, which was higher than the best accuracy reported in the related studies.

**Table 6:** Comparison of the accuracy in cross-subject experiments.

**Method**	**SEED**	**SEED-IV**
	**Mean/STD (%)**	**Mean/STD (%)**
DCORAL (Chai et al., 2016)	62.14/07.98	40.50/10.05
DANN (Ganin et al., 2016)	72.42/07.04	47.66/08.38
DAN (Li et al., 2018)	68.26/07.47	48.39/06.97
TANN (Li et al., 2021a)	84.41/09.18	68.00/08.35
BiHDM (Li et al., 2021b)	85.27/10.84	69.03/08.66
BiDANN (Li et al., 2021c)	84.14/06.87	65.59/10.39
MS-MDA (Chen et al., 2021)	80.62/11.03	57.92/10.12
GMSS (Li Y. et al., 2022)	76.04/11.91	62.13/11.91
UDDA (Li Z. et al., 2022)	88.10/06.54	73.14/09.43
MSMRA (Cao et al., 2022)	83.62/09.58	69.77/07.37
MS-ADA (She et al., 2023)	86.16/07.87	59.29/13.65
MFA-LR (Jiménez-Guarneros and Fuentes-Pineda, 2023)	85.27/10.84	69.58/14.10
Ours	**90.27/05.56**	**73.41/08.27**

Table 7 illustrates the results of the proposed SH-MDA and other advanced methods in the cross-session experiments. The results indicated that, among all the algorithms, the proposed model achieved optimal performance on both public datasets. On the SEED dataset, the average accuracy of the proposed model stood at 94.16%, surpassing the MS-ADA model's accuracy by ~3%. However, the classification performance generally decreased due to the increased difficulty in classification on the SEED-IV dataset. The average accuracy of the SH-MDA method was 75.05%, which denoted an obvious performance improvement of 2.67% compared with the other algorithms.

**Table 7:** Comparison of the accuracy in cross-session experiments.

**Method**	**SEED**	**SEED-IV**
	**Mean/STD (%)**	**Mean/STD (%)**
DCORAL (Chai et al., 2016)	78.86/07.06	44.63/11.38
DANN (Ganin et al., 2016)	90.42/09.64	63.07/12.66
DAN (Li et al., 2018)	80.00/08.88	58.36/12.77
MS-MDA (Chen et al., 2021)	89.60/07.20	65.89/10.12
MSMRA (Cao et al., 2022)	88.31/06.23	72.38/10.12
MS-ADA (She et al., 2023)	91.10/07.08	66.68/11.86
Ours	**94.16/06.92**	**75.05/08.19**

### 4.4 Confusion matrix

**Figure 8:**
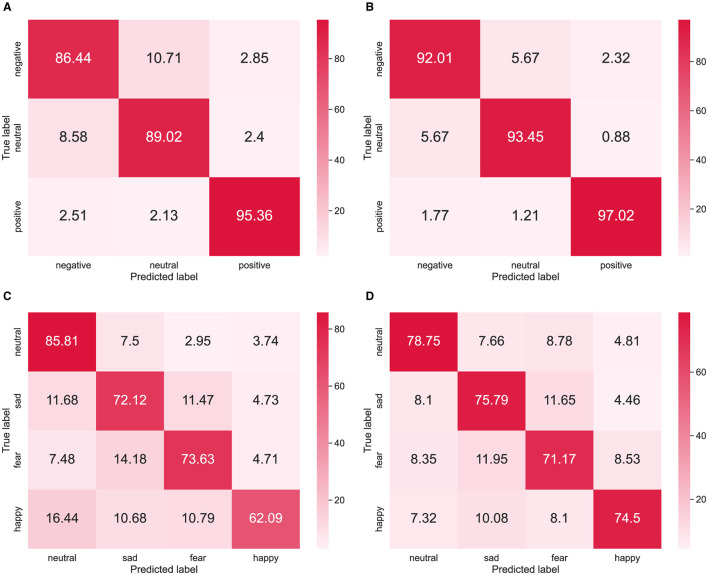
Confusion matrices of the SH-MDA in the cross-subject and cross-session experiments: **(A, B)** the results on the SEED dataset; **(C, D)** the results on the SEED-IV dataset.

The confusion matrices of the SH-MDA method for different datasets are presented in Figure 8. It should be noted that to ensure that the experimental results were convincing, the confusion matrix used data from three sessions. Figures 8A, B correspond to two different tasks on the SEED dataset. Obviously, in the cross-subject experiments, negative emotion was the most difficult to recognize, with an accuracy of 86.44%. In the two tasks, the model could easily confuse in the discrimination of neutral and negative emotions but maintained a high accuracy in the discrimination of positive emotions, with 95.36 and 97.02%.

Figures 8C, D show the confusion matrices on the SEED-IV dataset. The results indicated that the classification performance on the SEED-IV degraded compared with the SEED dataset. However, the model maintained good stability in recognizing each emotion category. In the two tasks,the model maintained high accuracy in the discrimination of neutral emotions, reaching 85.81 and 78.75%. Moreover, the model performed a high confusion probability in fear and sad emotion categories, which might indicate the potential relevance of the two emotions.

## 5 Discussion

### 5.1 Visualization

The t-SNE technology was used to visualize the changes of feature distribution of two dataset, which could reduce the data dimension while maintaining the data distribution in a low-dimensional space (Van der Maaten and Hinton, 2008). To simplify the experiment and enhance the visualization effects, the experiment was conducted only for the cross-subject task, and 100 EEG samples from each of the five subjects (domains) were randomly selected.

**Figure 9:**
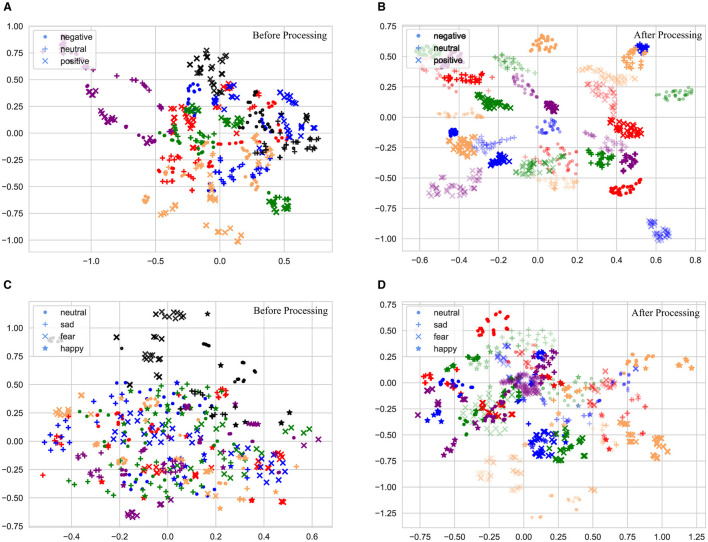
Scatter plot of feature distribution with the t-SNE on the SEED and SEED-IV datasets: **(A, B)** the feature distribution in the SEED dataset; **(C, D)** the feature distribution in the SEED-IV dataset.

The changes in feature distribution of the SEED and SEED-IV datasets through the model are illustrated in Figure 9. Different colors represent different domains, and different graphs are used to represent different emotion categories. Figures 9A–C show the original feature distributions. To highlight the distribution of target samples, the target domain samples before model processing are represented in black. The original feature distribution indicated that there was a clear difference between the source-domain samples and the target-domain samples. In addition, category confusion existed in both target and source domains, which manifested in the SEED dataset as confusion between negative and neutral emotions; this was consistent with the confusion mentioned in the earlier confusion matrix. In the SEED-IV dataset, category confusion indicates mutual confusion between various emotion categories, which explained the observed decline in performance in this dataset.

The feature distributions after processing by the proposed model are displayed in Figures 9B–D; for better visualization, the processed target-domain samples are denoted as translucent while maintaining the same colors as their corresponding source-domain data for easy distinction. The results indicated that, the source-domain and target-domain samples exhibited a concentration of samples from the same category and separation of those from different categories, exemplifying the excellent performance of the proposed model in multi-source domain adaptation tasks.

### 5.2 Ablation experiment

In this article, the ablation experiments were conducted on two datasets. In the cross-subject and cross-session experiments, the average value of the three sessions was used as the result, as shown in Table 8.

**Table 8:** The results of ablation experiments.

**Dataset**	**Method**	**ACC (mean** ±**std)**	
		**Cross-subject**	**Cross-session**
SEED	W/o ce loss	89.01 ± 6.11	92.87 ± 5.14
	W/o sample hybrid	86.32 ± 7.80	90.51 ± 6.71
	W/o dis loss	88.69 ± 8.14	93.1 ± 7.22
	W/o ce loss and sample hybrid	85.19 ± 6.97	89.21 ± 7.73
	**Full**	90.27 ± 5.56	94.16 ± 6.92
SEED-IV	W/o ce loss	71.64 ± 7.86	73.54 ± 11.92
	W/o sample hybrid	69.79 ± 8.86	72.91 ± 11.38
	W/o dis loss	72.46 ± 9.03	73.97 ± 10.45
	W/o ce loss and sample hybrid	68.05 ± 9.45	69.95 ± 9.91
	**Full**	73.41 ± 8.27	75.05 ± 8.19

The last row in Table 8 presents the performance of the complete proposed model. First, during the training process, the conditional entropy loss was ablated. With a relatively small decrease in accuracy of about 1%, it was demonstrated that pushing the decision boundary away from the high-density regions in the target domain contributed to the model performance improvement. Moreover, even without the conditional entropy loss, the proposed model's results outperformed most of the comparative methods.

There was a substantial drop in model accuracy upon ablation of the hybrid method, with decreases of 3.95 and 3.65% on the SEED dataset and 3.62 and 2.14% on the SEED-IV dataset. This decline may stem from the model's inability to account for the alignment of inter-domain conditional distributions. Subsequently, discrimination loss was ablated, which also leads to the decline of the model performance. Finally, when both the conditional entropy loss and the hybrid loss were removed, the model was forced to focus only on the classification and MMD losses, and there was a significant decrease in performance compared with the full model. This result further substantiated the superiority of the proposed model.

### 5.3 Limitations

Although this study has achieved good recognition results, there are still certain limitations. First, The hybrid sample set is obtained by hybridizing the samples from the source domain and the samples from the target domain according to the hybrid parameter λ, however, this paper only performs global hybridizing without considering each sample, so the selection of this parameter may not always be optimal for each sample. Second, by minimizing the classification loss, the hybrid samples were compelled to be classified into the category of their associated source-domain samples, thus indirectly enhancing the likelihood of the target-domain samples being transferred to the correct category. However, although most target domain samples could be transferred to the corresponding categories with the guidance of hybrid samples, a small number of samples failed to adapt effectively to the correct categories due to their low similarity with the source-domain samples. Moreover, although our method was evaluated on two datasets, its generalization capability maybe needs to be further studied. The comparisons in this paper were limited to single-center data with a relatively small scale. Currently, a substantial amount of data comes from multiple centers. And due to differences in collection equipment, stimulus materials, experimental design, and other factors, significant variations exist among data from different centers. The performance of our algorithm when dealing with cross-center data has not yet been validated and requires further study.

Therefore, future work should focus on developing adaptive sample hybridization methods that allow the weights and targets of sample hybridization to be adjusted adaptively based on the differences between different source and target domains. Additionally, to enhance the model's ability to recognize emotions across centers, it is important to actively explore domain generalization methods and combine them with existing domain adaptation models to further improve the model's generalization capability and practicality.

## 6 Conclusion

This study proposes a novel multi-source domain adaptation EEG emotion recognition network model named SH-MDA, which aims to alleviate the difficulties of emotion recognition from the limited generalization ability of EEG features across subjects and sessions. In addition, a sample hybridization method that can effectively achieve the alignment of conditional distributions between the source and target domains is introduced. The cross-subject and cross-session experiment results demonstrate that, in comparison to other advanced methods, the proposed method can exhibit superior adaptability to multiple source domains and achieve optimal results on two different databases. Furthermore, the results of the ablation experiments validate the effectiveness of the proposed approach, and the results of the t-SNE visualization indicate that the SH-MDA can separate target-domain samples with different labels and cluster samples with the same label, thus enhancing the recognition performance. We proposed a novel multi-source domain adaptation method for emotion recognition tasks across subjects and sessions. This method can also be applied to other EEG classification tasks based on domain adaptation, such as motor imagery and fatigue state classification. Although the proposed method performs well, there are still some limitations. For instance, the sample hybridization in the model relies on hybrid weights and lacks cross-center recognition capability. Therefore, in future work, the adaptive sample hybridization and domain generalization methods should be investigated to further improve the domain adaptation effectiveness and generalization ability of this model.

## Data Availability

Publicly available datasets were analyzed in this study. This data can be found here: https://bcmi.sjtu.edu.cn/home/seed/seed.html.
